# Transient Elastography and Controlled Attenuation Parameter (CAP) in the Assessment of Liver Steatosis in Severe Adult Growth Hormone Deficiency

**DOI:** 10.3389/fendo.2019.00364

**Published:** 2019-06-19

**Authors:** Adriana Claudia Lopes Carvalho-Furtado, Daniela Mariano Carvalho-Louro, Neysa Aparecida Tinoco Regattieri, Marcelo Palmeira Rodrigues, Maria Luiza Ricardo Nogueira Montenegro, André Metzker Ferro, Patrícia Souza Pirangi, Luciana Ansaneli Naves

**Affiliations:** ^1^Endocrinology Unit, Instituto Hospital de Base, Brasília, Brazil; ^2^Gastroenterology Unit, Instituto Hospital de Base, Brasília, Brazil; ^3^Radiology Unit, Faculty of Medicine, University of Brasilia, Brasília, Brazil; ^4^Pneumology Unity, Faculty of Medicine, University of Brasilia, Brasília, Brazil; ^5^Faculty of Medicine, University of Brasilia, Brasília, Brazil; ^6^Endocrinology Unit, Faculty of Medicine, University of Brasilia, Brasília, Brazil

**Keywords:** transient elastography, controlled atenuation parameter, liver steatosis, metabolic syndrome, adult growth hormone deficiency (AGHD)

## Abstract

Non-alcoholic fatty liver disease (NAFLD) is common in patients with growth hormone deficiency (GHD). Some noninvasive techniques have been used to quantify liver fat, such as the controlled attenuation parameter (CAP).

**Objective:** To evaluate CAP as a tool to identify liver steatosis and its relationship with different clinical and biochemical metabolic parameters in a group of patients with severe adult growth hormone deficiency (AGHD), and to compare the evolution of metabolic profiles after 6 months of human growth hormone (rhGH) replacement therapy in a subgroup of patients.

**Methods:** Cross-sectional observational study at baseline of naive rhGH multiple pituitary hormonal deficiency (MPHD) hypopituitarism patients. A 6-month intervention clinical trial in a selected group of a non-randomized, non-controlled cohort was also applied.

**Results:** Liver stiffness measurement (LSM) was normal in severe AGHD patients. CAP evaluation showed steatosis in 36.3% of baseline patients (8/22), associated with higher BMI, waist circumference, insulin, and alanine aminotransferase (ALT) levels. According to steatosis degree by CAP, child-onset growth hormone deficiency (CO-GHD) was graded as 68.75% (11/16) S0, 12.5% (2/16) S1, and 18.75% (3/16) S3, whereas AO-GHD was graded as 50% (3/6) S0, 16.66% (1/6) S2, and 33.33% S3. After 6 months of hrGH replacement, CAP measurements did not change significantly, neither on group without hepatic steatosis at baseline (194.4 ± 24.3 vs. 215.4 ± 51.3; *p* = 0.267) nor on the group with hepatic steatosis (297.2 ± 32.3 vs. 276.4 ± 27.8; *p* = 0.082). A significant improvement of body composition was observed only in the first group.

**Conclusions:** We have demonstrated the importance of CAP as a non-invasive tool in the liver steatosis identification on hypopituitary patients. This method may be an important indicator of the severity of metabolic disorders in MPHD patients. In our study, no liver health modification in LSM at baseline or after 6 months of rhGH replacement was found. Longer studies can help to establish the potential repercussions of growth hormone replacement therapy on liver steatosis.

## Introduction

Significant metabolic changes are observed in AGHD, such as negative effects on lipoprotein metabolism, lean mass reduction, fat mass increase, and intra-abdominal fat, leading to an increased risk of cardiovascular events (1–3). Central obesity and altered lipid profiles give rise to the development of Metabolic Syndrome (MetS), which has insulin resistance as a key pathogenic mechanism (4).

NAFLD is used to define the accumulation of liver fat in a patient without a prior history of alcohol abuse. The higher concentration of lipids, especially triglycerides in hepatocytes, leads to the development of hepatic steatosis, defined as the accumulation of fatty tissue higher than 5% of liver weight (5, 6).

Hepatic steatosis is considered a reversible change but may progress to an inflammatory process (steatohepatitis) and to liver cirrhosis. In this case, it may lead to a higher risk of hepatocarcinoma (7, 8). In addition, NAFLD represents an isolated cardiovascular risk factor and is associated with an increased incidence of cardiovascular disease (9).

The high prevalence of hepatic steatosis on AGHD patients, confirmed by liver biopsy, reveals the presence of an increased risk of progression to non-alcoholic steatohepatitis (NASH) (10). Therefore, the GHD in the subgroup of patients with MPHD represents a high risk of NAFLD and progression to NASH and cirrhosis (7, 11).

An important non-invasive technique has been used in the hepatology field to identify liver fibrosis—FibroScan® Echosens (12). Transient elastography (TE) by FibroScan® has been widely used in the last decade as a fast, non-invasive, and reproducible method to evaluate the liver stiffness measurement (LSM) as a measure of the degree of liver fibrosis. TE measures low frequency (50 Hz) elastic shear wave velocity propagation through the liver. In this case, shear wave velocity is directly related to tissue stiffness, which is expressed in kiloPascals (kPa). Usual tissue stiffness ranges from 2.5 to 7.5 kPa. A normal values for healthy liver stiffness is estimated to be about 5.5 kPa (13, 14). An available tool in FibroScan®, validated on patients with chronic liver disease from many causes, has enabled the evaluation of hepatic steatosis using controlled attenuation parameter (CAP). This parameter is useful for estimating the attenuation of the propagation of ultrasonic waves signals through the liver, acquired simultaneously with LSM by the FibroScan® probe. CAP is measured in decibels per meter (dB/m), with typical values ranging from 100 to 400 dB/m. CAP analysis is effective in the diagnosis and quantification of hepatic steatosis, from absolute cut-off points, with 90% sensitivity (15–18). Recent studies have shown that transient elastography has the best performance on the diagnosis and exclusion of advanced hepatic fibrosis in NAFLD (19). There is no previous literature report on the use of Transient Elastography to identify liver disease on MPHD hypopituitary patients.

## Objectives

The aim of this study is to evaluate the relationship of CAP with different clinical and biochemical metabolic parameters in a group of patients with severe AGHD. A secondary objective is to compare the evolution of metabolic profiles after 6 months of growth hormone replacement (rhGH) therapy in a selected group of patients.

## Subjects and Methods

### Study Design

This is a cross-sectional observational study at baseline of naive rhGH MPHD hypopituitarism patients and a 6-month intervention clinical trial in a selected group of a non-randomized, non-controlled cohort. Data were gathered and individuals were recruited from October 2016 thru October 2018 at the Endocrinology Unit of University Hospital of Brasilia, and at the Endocrinology Unit of Instituto Hospital de Base in Brasilia, located in the Federal District of Brazil.

Twenty-two patients were recruited at baseline and thirteen of them were enrolled in the intervention subgroup. From the intervention subgroup, patients who survived malignancies and those patients who did not agree to continue the study, were excluded from the group.

The study complied with the WMA Declaration of Helsinki and its amended versions on ethical principles for medical research involving human subjects and was approved by the Ethical Committee on Human Subject Research from the Fundação de Ensino e Pesquisa em Ciências da Saúde (FEPECS). All patients signed a proper informed consent before participating in the study.

### Inclusion Criteria

Patients included in the study were adults, with periodical ambulatory follow-ups, presenting at least one of the following criteria: (i) confirmed MPHD diagnosis; (ii) GHD initiated and treated in infancy or with disability developed in adulthood, for at least 5 years without rhGH replacement. GH deficiency was defined by GH peak < 3 ng/dl in the insulin tolerance test and IGF-I concentration <-2SD. Patients with hypopituitarism secondary to treatment of functioning pituitary tumors (Cushing's disease, prolactinoma, acromegaly); and chronic use of supraphysiological doses of corticoids were also excluded.

### Evaluation at Baseline

Variables considered were age, gender, signs, and symptoms at diagnosis. Also considered were comorbidities, anthropometric measurements, metabolic parameters (glucose, insulin, HOMA index, glycated hemoglobin, lipid metabolism, TGO, TGP, GGT) body composition by DXA, and hepatic evaluation by Fibroscan® with LSM and CAP measurement. All assessments were performed after night fasting.

#### Laboratory Analysis

Serum GH levels were measured by a two-site chemiluminescent immunometric assay (Immulite, 2000). Assay sensitivity was 0.01 ng/ml and the interassay coefficient of variation was <10%. Serum IGF-I was measured by a solid-phase enzyme labeled chemiluminescent immunometric assay with sample pretreatment on an onboard dilution step (Immulite, 2000). The manufacturer's normal range for age and gender was considered. Inter-assay coefficients of variation were <5%. Peptides were determined by chemiluminescent methods. Glucose, lipids, and transaminases were determined respectively by hexokinase and by IFCC without pyridoxal phosphate.

#### Body Composition by Dual Emission X-ray Absorptiometry (DEXA)

Body composition [including percent of fat body mass (FBM), truncal fat mass (TFM), lean body mass (LBM), and waist/hip ratio] was assessed by dual-emission X-ray absorptiometry (DXA, GE Healthcare, Lunar DPX NT, Diegem, Belgium) with participants in supine position (20).

#### FibroScan® Echosens, Paris, France

Transition Elastography (TE) was performed by a trained operator on the right lobe of the liver through the intercostal spaces with the patient lying in the dorsal decubitus position and with the right arm in maximal abduction. Ultrasound attenuation (CAP) was only calculated when the liver stiffness measurement (LSM) was valid in order to ensure an accurate attenuation. A reliable LSM was defined using the following 3 criteria: (i) at least 10 valid shots; (ii) a success rate (SR: the ratio of valid shots to the total number of shots) of at least 60%; and (iii) an interquartile range (IQR) of <30% of the median LSM (IQR/M\30%). The median value of 10 successful measurements was selected as the representative value. TE results were expressed as kilopascals (kPa) for LSM and as dB/m for CAP. LSM ranges from 2.5 to 7.5 kPa, and a healthy liver normal value is estimated to be 5.5 kPa (14, 21). The intensity of hepatic steatosis is graded by CAP according to published cut-offs bounds (for the M probe S0 <248; S1 = 248–267; S2 = 268–279; S3 ≥ 280 dB/m) (17).

### Intervention Subgroup

Patients in the intervention subgroup started rhGH replacement therapy at a dose of 0.2 mg/day (0.6UI) for men and 0.3 mg/day (0.9UI) for women on oral estrogen therapy. They were evaluated monthly, with a dose increase of 0.1 mg/day rhGH until reaching IGF-1 concentrations between −1SD and +1SD for sex and age. After 6 months, with optimal concentrations of IGF-1 reached, the initial evaluations were repeated. The same radiological and biochemical parameters were evaluated at baseline and after 6 months of treatment.

The recombinant growth hormone used for treatment was Hormotrop®(4IU, Bergamo). Patients with MPHD who had survived malignancies were excluded from this group.

### Statistical Analysis

The Kolmogorov-Smirnov and the Shapiro-Wilk tests were used to test the normality of the variables. The Pearson correlation test was used for normal variables, and the Spearman correlation test was used for non-normal variables. Student's *t*-test was used to analyze the significance of the variables with normal distribution, being expressed as media and standard deviation. The independent variables HOMA IR, IGF-I, insulin, triglycerides, truncal fat, ALT, and AST were considered non-normal and evaluated by the non-parametric Mann-Whitney test, being expressed as a median and interquartile interval. The Fisher exact test was used to test the association between two independent nominal variables.

Data analysis was performed using the independent variables before and 6 months after treatment with rhGH and was conducted using the *T*-test of paired samples for normal distribution variables and non-parametric tests of two independent samples for non-normal variables.

## Results

### Clinical Characteristics at Baseline

At baseline evaluation, the group of 22 patients presented a similar distribution between gender, and most of the patients had a diagnosis of GH deficiency in early childhood. Benign tumoral diseases were the most common etiology for hypopituitarism (36.4%), but inflammatory and malignant conditions were also observed. Half of the patients were submitted to a neurosurgical procedure. Six patients (27% of total) underwent previous adjuvant radiotherapy treatment of baseline pathology for more than 10 years (four patients with craniopharyngioma, one patient with medulloblastoma, and one patient with suprasellar dysgerminoma). ACTH deficiency was observed in 77% of the patients, treated by 5 mg of prednisone for more than 1 year (Table 1). From our sample, only one woman, 20 years old, had a normal gonadotropic axis. All hypogonadic patients (21/22) were under steroid replacement (estrogen/ progesterone or testosterone).

**Table 1:** Clinical aspects of baseline adult GHD patients.

**Characteristics**	**N^**°**^ (22)**	**% (100)**
Gender		
Male	11	50
Female	11	50
Diagnosis		
Childhood	16	72.7
Adulthood	6	27.3
Etiology		
Idiopathic	5	22.7
Craniopharyngioma	4	18.2
NFPA^a^	4	18.2
TBI^b^	2	9.1
Others^c^	7	31.8
Corticotherapy		
No	5	22.7
Yes	17	77.3
Time of GH deficiency		
5y−10y	13	59.1
>10y	9	40.9
Radiotherapy		
Yes	6	27.3
No	16	72.7
Neurosurgery		
Yes	11	50
No	11	50

a Non Functioning Pituitary Adenoma

b Traumatic Brain Injury

c 1- Sheehan Syndrome, 1-Hipoxic-ischaemic encephalopathy neonatal syndrome, 1-Meduloblastoma, 1-Dysgerminoma, 1-Astrocytoma, 1-Empty Sela, 1-Histiocytose

Patients who presented adult-onset growth hormone deficiency (AO-GHD) had higher BMI, waist circumference, waist/hip ratio, and presented higher HOMA-IR and insulin levels. Body fat distribution assessed by DXA body composition did not differ between groups (Table 2).

**Table 2:** Metabolic characteristics of baseline GHD patients according to deficiency onset phase.

	**Adult Onset**	**Child Onset**	***P-value***
	**(*n* = 6)**	**(*n* = 16)**	
Age (year)	44.13 ± 9.5	27.65 ± 8.0	0.000
BMI (kg/m^2^)	28.6 ± 3.5	23.2 ± 4.7	0.011
WC (cm)	98.7 ± 9.8	85.0 ± 12	0.021
LBM (%)	58.0 ± 7.9	58.6 ± 8.9	0.867
FBM (%)	39.3 ± 6.8	35.8 ± 8.9	0.403
TFM (%)	43.4 ± 5.0	45.4 ± 12	0.341
waist/hip ratio	1.2 ± 0.07	1.0 ± 0.10	0.003
IGF-1 (ng/ml)	70.5 ± 56.0	58.0 ± 48.0	0.494
HOMA IR	2.5 ± 2.3	0.7 ± 1.4	0.027
Glucose (mg/dl)	81.37 ± 8.9	77.8 ± 10.6	0.418
Insulin (μUI/ml)	12.7 ± 11.0	4.9 ± 6.2	0.049
HbA1c (%)	5.4 ± 0.3	4.8 ± 1.0	0.122
Total cholesterol (mg/dl)	185.4 ± 36.5	191.8 ± 48.7	0.743
LDL-cholesterol (mg/dl)	103.7 ± 30.3	112.1 ± 18.9	0.401
HDL-cholesterol (mg/dl)	48.9 ± 11.1	45.9 ± 10.0	0.510
Triglycerides (mg/dl)	134.5 ± 147	99.0 ± 127.5	0.407
LSM (kPa)	4.3 ± 0.9	4.8 ± 1.6	0.476
CAP (dB/m)	255.2 ± 56.1	228.3 ± 65.9	0.388

*BMI, body mass index; WC, waist circumference; LBM, lean body mass; FBM, fat body mass; TFM, truncal fat mass; IGF-1, insulin growth factor type 1; HOMA IR, insulin resistance index; HbA1c, glycated hemoglobin; LDL, low density lipoprotein; HDL, high density lipoprotein; LSM, liver stiffness measurement; CAP, controlled attenuation parameter*.

### Hepatic Evaluation by Elastography

Of the 23 patients submitted to FibroScan®, only one patient failed the method due to difficulty in acquiring valid measurements due to narrow intercostal spaces. The patient was excluded from the study. Considering FibroScan® results, LSM has not shown abnormalities or differences on GHD patients according to the deficiency onset phase, while absolute CAP values were higher in adult-onset patients but without statistical significance (Table 2). CAP evaluation showed hepatic steatosis in 36.3% of the patients (8/22), associated with higher BMI, waist circumference, insulin, and ALT. Lipid profile and body composition were not related to fatty liver disease identification by CAP (Table 3).

**Table 3:** Metabolic profile of baseline DGHA patients according to hepatic steatosis measured by CAP.

	**Hepatic Steatosis (CAP)**		
	**No**	**Yes**	**Valor *P***
	**(*n* = 14)**	**(*n* = 8)**	
Age (year)	31.6 ± 12.2	30.9 ± 11.7	0.897
Gender (M/F)	6/8	5/3	
Corticotherapy (N/Y)	3/11	2/6	
BMI (kg/m^2^)	21.6 ± 4.3	27.9 ± 3.9	0.003
WC (cm)	83.2 ± 11.2	98.2 ± 9.8	0.005
HOMA IR	0.75 ± 1.4	1.9 ± 1.9	0.110
Insulin (μUI/ml)	4.43 ± 7.1	8.5 ± 8.4	0.042
HbA1c (%)	5.1 ± 0.33	5.0 ± 0.33	0.458
IGF-1 (ng/ml)	52.0 ± 31.0	73.0 ± 61.2	0.330
Total cholesterol (mg/dl)	178.4 ± 22.1	190.0 ± 30.3	0.314
HDL-cholesterol (mg/dl)	48.1 ± 10.3	41.9 ± 12.4	0.218
LDL-cholesterol (mg/dl)	108.6 ± 22.9	100.2 ± 34.3	0.500
Triglycerides (mg/dl)	95.5 ± 80.0	164.5 ± 371.0	0.127
AST (U/L)	21.0 ± 8.0	24.0 ± 34.0	0.868
ALT (U/L)	16.0 ± 10.0	26.0 ± 29.0	0.010
GGT (U/L)	16.5 ± 13.0	45.0 ± 13.0	0.059
LBM (%)	59.3 ± 9.0	56.8 ± 7.8	0.516
FBM (%)	35.6 ± 9.0	39.0 ± 7.3	0.374
TFM (%)	43.0 ± 13.5	44.3 ± 3.9	0.815
waist/hip ratio	1.05 ± 0.10	1.08 ± 0.13	0.536
LSM (kPa)	4.6 ± 1.7	4.8 ± 0.9	0.721
CAP (dB/m)	196.1 ± 32.7	304.9 ± 37.5	0.001

*M/F, male/female; N/Y, yes/no; BMI, body mass index; WC, waist circumference; HOMA IR, insulin resistance index; HbA1c, glycated hemoglobin; IGF-1, insulin growth factor type 1; HDL, high density lipoprotein; LDL, low density lipoprotein; AST, aspartate aminotransferase; ALT, alanine aminotransferase; GGT, gamma glutamyltranspeptidase; LBM: lean body mass; FBM, fat body mass; TFM, truncal fat mass; LSM, liver stiffness measurement; CAP, controlled attenuation parameter*.

**Figure 1:**
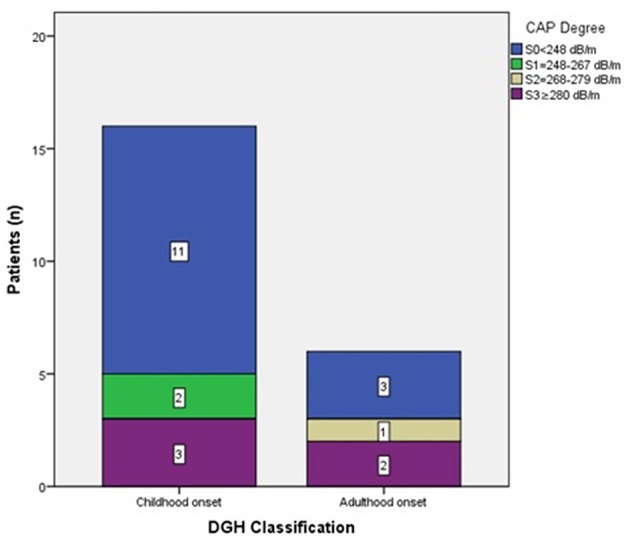
patients number according CAP degree.

According to steatosis degree by CAP, child-onset growth hormone deficiency (CO-GHD) was graded as 68.75% (11/16) S0, 12.5% (2/16) S1, and 18.75% (3/16) S3, whereas AO-GHD was graded as 50% (3/6) S0, 16.66% (1/6) S2, and 33.33% S3 (Figure 1).

**Figure 2:**
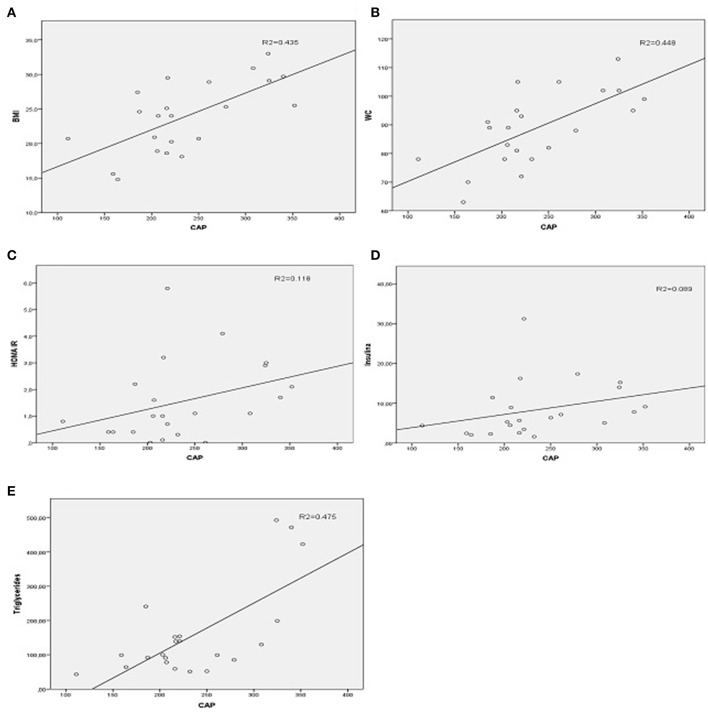
Spearman correlation graphics between CAP and metabolic parameters. **(A)** CAP, controlled attenuation parameter(dB/m) and BMI, body mass index (kg/m^2^). **(B)** CAP and WC, waist circumference (cm). **(C)** CAP and HOMA IR, homeostatic model assessment insulin resistance. **(D)** CAP and Insulin (μUI/ml). **(E)** CAP and Triglycerides (mg/dl).

In order to test the relationship between CAP and the independent variables of metabolic syndrome, the Spearman correlation was used (Figure 2). Moderately positive correlation results were found between CAP measurements with BMI (ρ = 0.621; *p* = 0.002), WC (ρ = 0.632; *p* = 0.002), HOMA IR (ρ = 0.447; *p* = 0.037), insulin (ρ = 0.515; *p* = 0.014), and triglycerides (ρ = 0.476; *p* = 0.025).

### Clinical Characteristics of Therapeutic Intervention Subgroup

Patients in the intervention subgroup started rhGH replacement therapy at a dose of 0.2 mg/day (0.6UI) for men and 0.3 mg/day (0.9UI) for women on oral estrogen therapy.

After 6 months of rhGH replacement therapy, the patients showed significant improvement in body composition, with a reduction of waist circumference and fat body distribution. A significant gain of lean body mass and reduction of fat body mass were also observed, and there was no change in BMI. However, a significant increase in blood glucose, insulin, and HOMA-IR index were observed. LSM and CAP measurements did not change significantly (Table 4).

**Table 4:** Metabolic changes in the intervention group after 6 months of rhGH replacement therapy.

	**Baseline (*n* = 13)**	**After 6 months (*n* = 13)**	***P-value***
BMI (kg/m^2^)	24.03 ± 6.0	24.43 ± 5.0	0.360
WC (cm)	89.4 ± 14.1	87.0 ± 12.3	0.034
IGF-I (ng/ml)	50.0 ± 45.0	169.0 ± 64.5	0.002
Glucose (mg/dl)	79.1 ± 8.3	85.5 ± 6.2	0.004
Insulin (μUI/ml)	5.1 ± 12.1	7.2 ± 11.3	0.013
HOMA IR	1.1 ± 2.6	1.5 ± 2.6	0.006
HbA1c (%)	5.2 ± 0.2	5.2 ± 0.2	0.331
Total cholesterol (mg/dl)	185.8 ± 30.2	186.8 ± 41.2	0.919
HDL-cholesterol (mg/dl)	47.7 ± 11.6	42.8 ± 8.1	0.059
LDL-cholesterol (mg/dl)	110.0 ± 27.0	99.2 ± 25.3	0.251
Triglycerides (mg/dl)	99.0 ± 102.0	139.0 ± 276.0	0.064
AST (U/L)	23.0 ± 8.0	23.0 ± 6.0	0.753
ALT (U/L)	21.0 ± 13.0	16.0 ± 18.0	0.937
GGT (U/L)	17.0 ± 22.0	19.0 ± 23.0	0.700
CAP (dB/m)	217.0 ± 108.0	245.0 ± 98.0	0.972
LSM (kPa)	5.07 ± 1.62	4.62 ± 1.25	0.181
LBM (%)	60.4 ± 8.5	64.7 ± 10.7	0.004
FBM (%)	34.7 ± 8.8	32.0 ± 9.8	0.008
TFM (%)	43.9 ± 5.9	41.7 ± 15.5	0.039
waist/hip ratio	1.10 ± 0.18	1.06 ± 0.13	0.021

*BMI, body mass index; WC, waist circumference; IGF-1, insulin growth factor type 1; HOMA IR, insulin resistance index; HbA1c, glycated hemoglobin; HDL, high density lipoprotein; LDL, low density lipoprotein; AST, aspartate aminotransferase; ALT- alanine aminotransferase; GGT, gamma glutamyltranspeptidase; CAP, controlled attenuation parameter; LSM, liver stiffness measurement; LBM, lean body mass; FBM, fat body mass; TFM, truncal fat mass*.

Intervention subgroup patients were divided according to the presence of hepatic steatosis measured by CAP, and a statistically significant improvement on body composition was observed only in patients without hepatic steatosis after 6 months of hrGH replacement therapy. CAP measurements did not change significantly, neither on the group without hepatic steatosis (194.4 ± 24.3 vs. 215.4 ± 51.3; *p* = 0.267) nor on the group with hepatic steatosis (297.2 ± 32.3 vs. 276.4 ± 27.8; *p* = 0.082), during 6 months of follow up. However, worsening absolute values of glucose, insulin, and HbA1c were found in the group with hepatic steatosis at baseline, despite the WC reduction (Table 5).

**Table 5:** Metabolic changes in the intervention group after 6 months of rhGH replacement therapy according to hepatic steatosis measured by CAP.

	**HEPÁTIC STEATOSIS**					
	**NO (<248 dB/m)**			**YES (≥248 dB/m)**		
	**Baseline (*****n*** **=** **8)**	**After 6 months (*****n*** **=** **8)**	***P-value***	**Baseline (*****n*** **=** **5)**	**After 6 months (*****n*** **=** **5)**	***P-value***
Gender (M/F)	5/3			3/2		
Corticotherapy (Y/N)	7/1			3/2		
rhGH dose (mg)	0.68 ± 0.2			0.48 ± 0.2		
BMI (kg/m^2^)	21.7 ± 5.5	22.4 ± 4.2	0.296	27.8 ± 4.9	27.7.0 ± 4.65	0.704
WC (cm)	84.4 ± 13.3	*83.0*±11.4	0.390	97.4.0 ± 12.4	93.4 ± 11.9	< 0.001
IGF-I (ng/ml)	43.5 ± 13.6	158.1 ± 38.5	< 0.001	87.6 ± 38.7	182.1 ± 40.8	0.005
Glucose (mg/dl)	76.6 ± 7.2	82.7 ± 4.3	0.058	83.2 ± 9.2	89.8 ± 6.6	0.039
Insulin (μUI/ml)	5.6 ± 5.3	7.5 ± 2.7	0.147	11.6 ± 5.5	18.0 ± 9.0	0.024
HOMA IR	1.05 ± 1.1	1.5 ± 0.6	0.073	2.4 ± 1.3	4.1 ± 2.2	0.026
HbA1c (%)	5.1 ± 0.25	5.2 ± 0.18	0.096	5.2 ± 0.1	5.3 ± 0.2	0.056
Total cholesterol (mg/dl)	179.5 ± 26.0	170.1 ± 35.8	0.474	195.8 ± 36.8	213.4 ± 37.5	0.262
HDL-cholesterol (mg/dl)	49.6 ± 11.8	45.6 ± 6.6	0.305	44.6 ± 11.8	38.2 ± 9.0	0.060
LDL-cholesterol (mg/dl)	107.1 ± 28.8	96.0 ± 21.0	0.184	114.5 ± 26.0	104.4 ± 33.2	0.662
Triglycerides (mg/dl)	117.4 ± 59.8	150.5 ± 126.3	0.426	191.8 ± 177.2	367.0 ± 317.3	0.085
AST (U/L)	27.0 ± 8.4	24.7 ± 6.6	0.526	21.0 ± 4.2	26.2 ± 8.8	0.308
ALT (U/L)	20.1 ± 10.1	18.5 ± 9.8	0.756	26.0 ± 9.6	31.8 ± 17.6	0.502
GGT (U/L)	18.6 ± 11.0	15.9 ± 8.3	0.339	55.4 ± 62.3	69.8 ± 70.7	0.433
LSM (kPa)	5.3 ± 1.9	4.7 ± 1.5	0.294	4.7 ± 1.02	4.4 ± 0.9	0.354
CAP (dB/m)	194.4 ± 24.3	215.4 ± 51.3	0.267	297.2 ± 32.3	276.4 ± 27.8	0.082
LBM (%)	62.0 ± 10.2	67.4 ± 12.9	0.016	57.72 ± 4.5	60.2 ± 4.0	0.152
FBM (%)	32.5 ± 10.5	28.6 ± 11.2	0.012	38.2 ± 3.5	37.5 ± 3.2	0.234
TFM (%)	43.0 ± 11.7	32.8 ± 12.0	0.043	44.1 ± 1.6	48.4 ± 11.2	0.440
waist/hip ratio	1.05 ± 0.11	0.98 ± 0.10	0.060	1.14 ± 1.07	1.11 ± 0.07	0.274

*M/F, male/female; N/Y, yes/no; rhGH, human growth hormone; BMI, body mass index; WC, waist circumference; IGF-1, insulin growth factor type 1; HOMA IR, insulin resistance index; HbA1c, glycated hemoglobin; HDL, high density lipoprotein; LDL, low density lipoprotein; AST, aspartate aminotransferase; ALT- alanine aminotransferase; GGT, gamma glutamyltranspeptidase; LSM, liver stiffness measurement; CAP, controlled attenuation parameter; LBM: lean body mass; FBM, fat body mass; TFM, truncal fat mass*.

## Discussion

Transient elastography by Fibroscan has been used as a fast, non-invasive, and reproducible method to evaluate liver fibrosis (13, 14). In addition to accurately evaluating LSM, TE is also capable of quantifying hepatic steatosis through a physical parameter (CAP), which is simultaneously acquired with LSM by the FibroScan probe and which estimates the liver attenuation levels (17, 18).

CAP is very efficient in detecting even low-grade steatosis, but neither the presence nor the severity of hepatic steatosis as measured by CAP predicts liver-related events, cancer, or CVE in the short period of time (15, 17).

The positive correlation observed between CAP and BMI, WC, and insulin resistance markers in the sample used in this study confirms the important role of this non-invasive tool in the identification of NAFLD on hypopituitarism patients. This represents an independent predictor of CVD (22, 23). This finding may be an alert for screening clinical features used to detect early risk of developing metabolic syndrome. Considering criteria based on insulin resistance, and supported by waist circumference data, MetS prevalence ranges between 10 and 84% worldwide, depending on the geographic region, urban or rural environment, individual demographic characteristics of the population studied (gender, age, racial, and ethnic origin), and the criteria used to define MetS (24).

At baseline, BMI was higher in adult-onset GH deficiency, but both groups presented a high percentage of fat mass, suggesting that there is a negative repercussion of GH deficiency in body composition (25). Greater alteration of insulin resistance clinical profiles, such as BMI, WC, waist/hip ratio, HOMA IR, and insulin, was observed in the group of patients with AO-GHD compared to the CO-GHD group. As the interval (years) without rhGH replacement therapy was similar between the groups, the differences found on clinical parameters may be related to genetic predisposition and to exposure to general factors such as eating behaviors, dietary factors contributing to insulin resistance, nutrient availability, and reduced or absent physical activity (26–28).

Furthermore, aging is truly important as an insulin resistance combined factor related to changes in body composition, insulin activity suppression by substrates such as free fatty acids (FFAs), and likely resistance to leptin (29–31). Thereby, as discussed by other authors, CO-GHD patients are more prone to accumulated metabolic risks because they have a higher time forward to hormonal deficiency exposure (32).

Moderated hypercholesterolemia due to an increase in low-density lipoprotein (LDL) cholesterol has been reported in patients with GHD (25). These data were not observed in the studied sample and could be explained in part by the replacement of rhGH in the group of patients with CO-GHD during childhood. The use of statins (data not shown) and the extreme individual lipid variations (mainly in the concentration of triglycerides) also contributed to these findings.

At baseline, the IGF1 showed no significant difference between groups, contrary to the literature reports (33–35), and may be related to the severity of pituitary deficiency present in both groups of MPHD patients.

A general overview of pathophysiological mechanisms that lead to insulin resistance in GHD patients gives them a higher risk of NAFLD development (23). The evaluation of hepatic steatosis by CAP of the considered sample confirmed the presence of risk factors such as for overweight, increased WC, and increased insulin levels as components of MetS hepatic manifestation (36, 37).

Liver biopsy remains the gold standard for the diagnosis of steatohepatitis and staging of fibrosis in patients with NAFLD, which is the strongest predictor for disease-specific mortality. Nevertheless, liver biopsy is invasive and associated with a low rate of complications such as pain and bleeding and restrains liver disease monitoring (14, 38). The current European Association for the Study of the Liver (EASL) guidelines for the management of NAFLD recommend TE as a non-invasive method for liver fibrosis assessment and monitoring (39). A recent study confirmed that transient elastography has the best non-invasive performance to evaluate liver fibrosis in NAFLD (19). In our series, no fibrosis changes were suggested by LSM, so a liver biopsy was not indicated in this cohort. In MPHD hypopituitary patients, complication and risks have increased such as hemorrhage, related to the use of corticosteroids, estrogens, and testosterone (11).

Patient with liver steatosis could also present a higher concentration of alanine aminotransferase with or without steatohepatitis. Although CAP measures reached statically significant transaminase differences, the absolute values were not higher than the reference values. These findings are in agreement with reports of normal transaminase in more than 70% of the patients with NAFLD (40).

Considering the metabolic findings hold up the development of NAFLD in the AO-GHD group, we observed a prevalence of severe steatosis on CO-GHD patients, highlighting the need for early diagnosis and intervention in this GHD group.

Intervention with rhGH in a selected group of patients with GHD resulted in a statistically significant effect on the corporal composition profiles, with an increase of lean mass and decrease of fat mass, truncal fat mass, and waist/hip ratio. This only occurred within the group of patients without hepatic steatosis at baseline. The lack of improvement in corporal composition in patients with steatotic liver may suggest a reduction of GH signaling in this condition, as recently described by Rufinatscha (41). On the other hand, this hypothesis could also suggest that the non-improvement of liver steatosis degree measured by CAP in our study could be related to reduced GH action on hepatic enzymes important to lipidic metabolism. This finding is in agreement with reports of non-improvement of steatosis with rhGH on GHD patients measured by other steatosis identification methods (37). This is in disagreement with the important role of GH and IGF-1 in the liver and the NAFLD improvement (42) and could be related to the short period of follow up in our study.

The worsening of the insulin resistance observed on the intervention group associated with steatosis by CAP could be transitory and followed by recovery of insulin sensitivity after therapy suspension, as has already been established (43, 44). At the same time, it was recently reported that the GH signaling in the liver could be diminished in patients with NAFLD, leading to hepatic insulin sensitivity and metabolic activity deterioration (41).

In conclusion, we have demonstrated the importance of CAP as a non-invasive tool in liver steatosis identification of hypopituitary patients. This method may be an important indicator of the severity of metabolic disorders in MPHD patients. In our study, no liver health modification in LSM at baseline and after 6 months of rhGH replacement was found. Further studies can help to establish the potential repercussion of growth hormone replacement therapy on liver steatosis.

## Data Availability

The datasets generated for this study are available on request to the corresponding author.

## Ethics Statement

The study complied with the WMA Declaration of Helsinki and its amended versions on ethical principles for medical research involving human subjects and was approved by the Ethical Committee on Human Subject Research from Fundação de Ensino e Pesquisa em Ciências da Saúde (FEPECS). All patients signed a proper informed consent before participating in the study.

## Author Contributions

All authors listed have made a substantial, direct and intellectual contribution to the work, and approved it for publication.

### Conflict of Interest Statement

The authors declare that the research was conducted in the absence of any commercial or financial relationships that could be construed as a potential conflict of interest.
